# Research on the Design of an On-Line Lubrication System for Wire Ropes

**DOI:** 10.3390/s25092695

**Published:** 2025-04-24

**Authors:** Fan Zhou, Yuemin Wang, Ruqing Gong

**Affiliations:** 1 College of Power Engineering, Naval University of Engineering, Wuhan 430030, China; zf422725587@163.com (F.Z.); aoi97s@163.com (R.G.); 2 College of Intelligent Manufacturing, Wuhan Technical University, Wuhan 430079, China

**Keywords:** specialty grease, lubrication system, wire rope, kinematic modeling

## Abstract

This study presents an on-line intelligent lubrication system utilizing specialty grease to address lubricant loss and uneven coating issues in traditional methods. Characterized by scanning electron microscopy (SEM) and Fourier transform infrared spectroscopy (FT-IR), the specialty grease demonstrates superior tribological performance, achieving a 46.7% reduction in the average friction coefficient and 33.3% smaller wear scar diameter under a 392 N load compared to conventional lubricants. The system features an automatic control vehicle design integrating heating, grease supply, lubrication-scraping mechanisms, and a dual closed-loop intelligent control system combining PID-based temperature regulation with machine vision. Experiments identified 50 °C as the optimal heating temperature. Kinematic modeling and grease consumption analysis guided greasing parameters optimization, validated through simulations and practical tests. Evaluated on a 20 m long, 36.5 mm diameter wire rope, the system achieved full coverage within 60 s, forming a uniform lubricant layer of 0.3–1.0 mm thickness (±0.15 mm deviation). It realizes the innovative application of high-adhesion lubricating grease, adaptive process control, and real-time thickness feedback technology, significantly improving the lubrication effect, reducing maintenance costs, and extending the lifespan of the wire rope. This provides intelligent lubrication technology support for the reliable operation of wire ropes in industrial fields.

## 1. Introduction

Wire ropes are widely utilized in critical areas such as aerospace, hoisting and transportation, and marine engineering due to their high strength and wear resistance [1,2,3]. Serving as the core components of equipment, they play a vital role in ensuring system safety and operational stability. However, as service time increases, the protective layer on wire rope surfaces gradually degrades due to wear and corrosion, ultimately affecting their service life and safety [4,5]. Therefore, regular and effective lubrication and protection of wire ropes are particularly crucial [6,7,8,9,10,11,12,13,14,15]. Currently, the primary method for wire rope maintenance is greasing, where lubricants (such as lubricating oil or grease) are evenly applied to the wire rope surface through spraying, brushing, or rolling [16]. In this greasing process, since the lubricant is typically in a liquid state, issues like excessive lubricant dripping, uneven coating, and improper lubricant consumption often arise. These problems can lead to suboptimal wire rope lubrication and even compromise its safety and reliability. Specialty grease, an innovative lubricant material, offers a new solution for wire rope lubrication and protection due to its solid-state nature at room temperature. In contrast to liquid grease, specialty grease exhibits higher stability, stronger adhesion, and superior anti-corrosion performance. It can form a uniform and long-lasting lubrication film on the wire rope surface, effectively reducing friction and wear between the rope and its surrounding environment and preventing rust and corrosion. Additionally, specialty grease is less likely to be lost or drip during use, significantly reducing environmental pollution. Nevertheless, coating with specialty grease also poses certain challenges. Owing to its solid state at room temperature, it is difficult to achieve uniform and efficient coating using traditional methods. Therefore, developing an intelligent lubrication system based on specialty grease to achieve precise and efficient application of solid grease and enhance wire rope lubrication and protection is of great significance.

This paper focuses on an intelligent lubrication system for wire ropes using specialty grease, which differs from traditional wire rope greasing methods [17]. The research mainly encompasses aspects such as the optimization of grease formulations, the structural design of the lubrication system, and the development of the intelligent control system. The respective advantages and disadvantages are summarized, and improvement suggestions are proposed based on the existing lubrication system structure considering the application scenario of this study.

Recent research on optimizing grease formulations has shown promising results. Zhang et al. [18] introduced lanthanum stearate-modified lubricating grease (LSMLO) prepared via saponification reaction. Their findings revealed that when added to the base lubricant at 0.2 wt.%, LSMLO can significantly enhance wire rope lubrication. Compared to crude lubrication, it reduces the friction coefficient by approximately 35% and the wear diameter by about 25%, demonstrating superior anti-wear performance across different sliding speeds and loads. Zhang et al. [19] investigated α-ZrP-modified mining wire rope grease. They found that an appropriate amount of α-ZrP can decrease the friction coefficient, but an excessive amount may cause aggregation and degrade the lubrication effect. Principal component analysis indicated that α-ZrP with a mass fraction of 2.5% had the most pronounced positive impact on wire rope grease. Feng et al. [20] studied the rheological properties of friction-increasing grease and discovered that these properties significantly affect their frictional interaction with the wire rope’s friction lining. Xu et al. [21] added ore particles to grease and demonstrated that this can alter the micromotor behavior of helically structured wire ropes. These studies provide a scientific basis and practical methods for optimizing grease formulations to enhance wire rope friction performance and extend service life.

Existing research on wire rope maintenance systems has focused on optimizing greasing mechanisms to enhance operational efficiency and adaptability. Fang et al. [22] proposed a six-wheeled wire rope-climbing robot for sluice gate maintenance, which integrates wheeled and tracked locomotion advantages to achieve stable climbing and precise inspection capabilities. Similarly, Fu [23] developed a multi-stage decontamination device incorporating rough and semi-fine washing units, demonstrating effective dirt removal across varying rope diameters while ensuring smooth rope head passage. To address industrial demands for cost reduction, Madanhire et al. [24] designed an automated mining wire rope lubricator that combines pre-cleaning and lubrication functions, reportedly reducing manual labor by 50% and maintenance costs by 42%. Recent advancements include Wang et al. [25] designed a device to clean and lubricate wire ropes along the twisting direction of the rope strands. It addresses issues such as old grease accumulation and reduces weight and traction force requirements. Verified through kinetic modeling and experiments, the device has advantages like low traction force, stable speed, and good coaxially, meeting the maintenance needs of ropeway wire ropes. Further integration of smart technologies is exemplified by Chen et al. [26], who created an omnidirectional cleaning system featuring adaptive brush heads with pressure-regulated axial cleaning capabilities, achieving full-coverage maintenance without dead zones. These innovations collectively advance wire rope maintenance through structural optimization, process automation, and performance validation, though limitations persist in adaptive greasing control and long-term stability under variable operational conditions.

To further enhance the intelligence level of the lubrication system, researchers are dedicated to developing advanced intelligent control systems. Gao et al. [27] proposed an integrated control system scheme for wire rope cleaning and inspection lines based on PLC and touch screen technology. The system achieves automation control through PLC and touch screen interaction. PLC controls the device via I/O, and the touch screen communicates with PLC through RS232. This system enables manual and automatic control and temperature control functions, enhancing automation and intelligence levels, real-time parameter monitoring, and production efficiency. Belmas et al. [28] studied the non-destructive monitoring of steel cable stress states using electrical signals. They found that resistance changes reflect the integrity of steel cables, constructed mathematical models to analyze stress current distributions, and proposed using electrical signals to diagnose steel cable states to improve suspension bridge safety and lifespan.

In summary, although significant progress has been made in lubricating grease selection and lubrication system design, there are still limitations. Current grease formulation optimizations are mostly conducted under laboratory conditions, and their long-term stability and environmental friendliness in complex industrial environments require further verification. Despite the diverse designs of lubrication systems, their adaptability needs improvement, especially in responding to different working conditions and automatically adjusting the greasing consumption, thickness, and uniformity. This study aims to design an intelligent lubrication system for wire ropes based on specialty grease to address the issues of specialty grease coating in existing lubrication systems. By focusing on the physicochemical properties of specialty grease, combined with the actual use of wire ropes, and through theoretical calculations, simulations, and experimental validations, this paper will conduct in-depth research on the structure of the lubrication system, the control system, and the greasing process. The goal is to enhance the lubrication and maintenance effects, extend the service life of wire ropes, and reduce maintenance costs and safety risks. Meanwhile, this study will also offer valuable references for the application of intelligent greasing technology in other fields.

## 2. Composition and Physicochemical Properties of Specialty Grease

Wire ropes endure extreme operational stresses, including high tension, dynamic bending, and exposure to corrosive environments. Effective lubrication necessitates grease capable of firm adhesion to resist mechanical displacement and environmental wash-off, uniform penetration into wire interstices to prevent localized wear, and thermal resilience across −20 °C to 120 °C to maintain structural stability. The grease must exhibit anti-wear and extreme-pressure properties under loads to reduce friction and galling, while corrosion resistance safeguards against moisture, salt, and chemical degradation. Controlled temperature-dependent viscosity ensures fluidity during application and solid stability at ambient conditions to minimize dripping. Environmental compliance further requires low volatility and non-toxicity to align with industrial regulations. These criteria collectively define the targeted performance framework for wire rope lubrication systems.

### 2.1. Spectral Characterization of Specialty Grease

**Figure 1:**
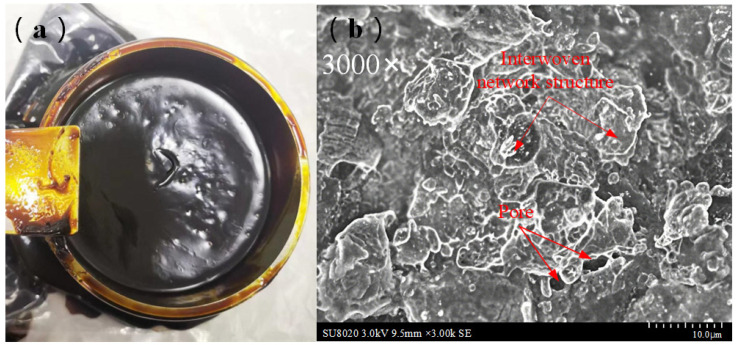
SEM images of the specialty grease microstructure: (**a**) the actual state of specialty grease; (**b**) three-dimensional interwoven network structure of the calcium-based soap thickener and high-magnification view showing physical adsorption of base oil and additives within the network pores (scale bar: 10 μm).

Grease is typically composed of base oil, additives, and a soap thickener (metallic soap). The base oil, derived from mineral or synthetic oils, provides excellent lubricity. Additives are incorporated to enhance specific properties; for instance, 5% nano-graphite or borate extreme-pressure additives can improve anti-wear performance. The soap thickener acts as a “sponge” to physically adsorb the base oil and additives, forming a stable colloidal structure. The specialty grease studied herein exists in a solid state at ambient temperature, attributed to its highly crystalline composite thickener structure (metallic soap content: 18–22%). Scanning electron microscopy (SEM) revealed a three-dimensional interwoven network structure (Figure 1), which immobilizes the base oil and additives within its pores via physical adsorption, thereby stabilizing the colloidal system, as shown in Figure 1.

**Figure 2:**
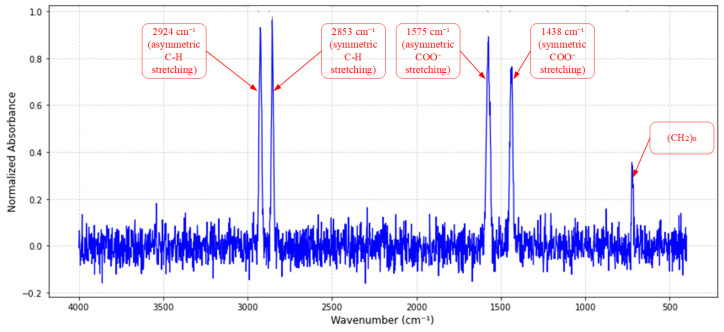
FT-IR analysis of the specialty grease: key absorption peaks: 1575 cm^−1^ (asymmetric COO^−^ stretching), 1438 cm^−1^ (symmetric COO^−^ stretching), and 1230 cm^−1^ (S=O vibration of sulfonate additives). The Δv value (137 cm^−1^) confirms calcium soap crystallization, while the base oil is identified as iso-alkane synthetic hydrocarbon (C–H stretching at 2924/2853 cm^−1^).

Fourier transform infrared spectroscopy (FT-IR) analysis (Figure 2) identified strong absorption peaks at 1575 cm^−1^ and 1438 cm^−1^, corresponding to the asymmetric and symmetric stretching vibrations of the carboxylate group (COO^−^) in metallic soap, respectively. The peak separation (Δv = 137 cm^−1^, calculated via Equation (1)) aligns with the crystal field splitting pattern of calcium-based soap.

(1) $$\Delta v=v_{asym}\left({COO}^{-}\right)-v_{sym}\left({COO}^{-}\right)=1575-1438=137\;{cm}^{-1}$$

The base oil component exhibited C–H asymmetric/symmetric stretching vibrations at 2924 cm^−1^ and 2853 cm^−1^ (half-peak width Δν = 15 cm^−1^), indicative of highly linear alkane chains. Combined with the CH_3_ bending vibration at 1377 cm^−1^, the base oil was inferred to be an iso-alkane type synthetic hydrocarbon (60–65% content). A planar rocking vibration peak at 720 cm^−1^ [(CH_2n_) ≥ 4] further confirmed its long-chain structure, which endows the base oil with excellent high-temperature oxidation stability and boundary lubrication capabilities. Notably, the broad peak at 1230 cm^−1^ likely originates from the S=O vibration of sulfonate additives, corroborating the low wear scar diameter (0.48 mm, Table 1) observed in four-ball tests. This suggests that sulfur/phosphorus additives form protective FeS/FePO_4_ films on metal surfaces through tribochemical reactions, enabling the grease to achieve both high permeability and superior anti-wear performance.

### 2.2. Performance Analysis of Specialty Grease

#### 2.2.1. Four-Ball Friction Tests

**Figure 3:**
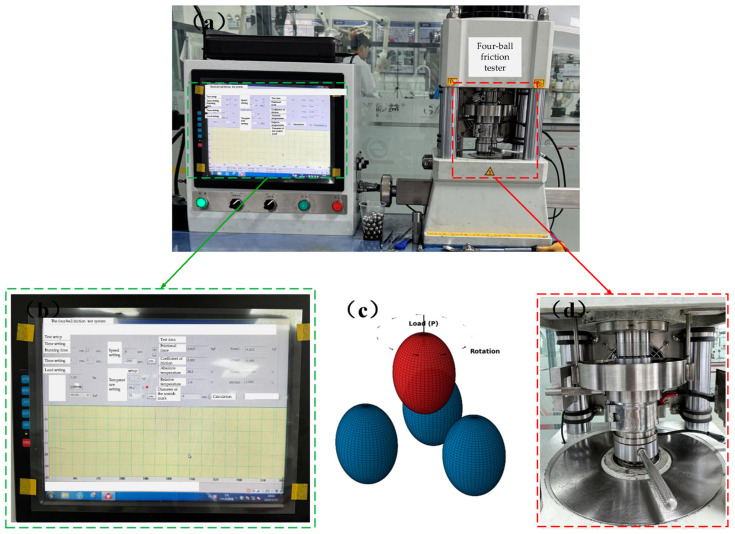
Tribological performance comparison between specialty and conventional greases: (**a**) four-ball friction tester; (**b**) parameters of four-ball friction tests; (**c**) four-ball friction test schematic diagram; (**d**) four-ball friction test mechanism.

The tribological properties of the specialty grease and conventional grease were evaluated using an MS-10A four-ball friction tester (schematic diagram shown in Figure 3) under the test conditions outlined in Table 1. The test parameters were set as follows: spindle speed of 1200 rpm, temperature of 75 °C, stepwise loading up to 392 N, and a duration of 60 min. The wear scar diameter (WSD) of the lower test ball was measured using white light interferometry, and real-time friction coefficient monitoring was employed to systematically assess the extreme pressure (EP) and anti-wear characteristics of both greases.

**Table 1:** Parameters of four-ball friction tests.

Test Parameters	Load (N)	Spindle Speed (r/min)	Time (s)	Temperature (°C)
Extreme pressure test	/	1450	10	50
Friction and wear test	392	600	3600	75
	392	1200	3600	75
	392	1800	3600	75

**Figure 4:**
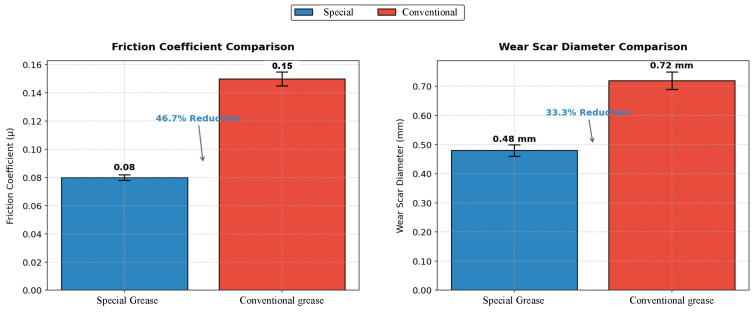
Comparative friction coefficients and wear scar diameters of the tested greases.

The experimental results (Figure 4) demonstrate the superior tribological performance of the specialty grease. The superior tribological performance of the specialty grease arises from its advanced formulation and structural characteristics. As demonstrated by FT-IR analysis, sulfur-phosphorus additives in the grease undergo tribochemical reactions under high loads, forming protective FeS/FePO_4_ films on metal surfaces. These films minimize direct metal-to-metal contact, thereby reducing friction and wear. Additionally, the three-dimensional interwoven network structure observed via SEM (Figure 1) effectively immobilizes the base oil and additives through physical adsorption, ensuring stable lubrication under shear stress. This structural integrity prevents oil separation and maintains a continuous lubricating film, contributing to the 46.7% reduction in friction coefficient compared to conventional grease. Wear morphology analysis revealed a wear scar diameter of 0.48 ± 0.02 mm for the specialty grease, significantly smaller than the 0.72 ± 0.05 mm observed for the conventional grease, corresponding to a 33.3% reduction in wear volume. To evaluate the influence of operational speed on grease performance, additional four-ball tests were conducted at spindle speeds ranging from 600 rpm to 1800 rpm under a constant load of 392 N. Results indicated that the specialty grease maintained a stable friction coefficient (0.08 ± 0.01) and wear scar diameter (0.48 ± 0.03 mm) across all tested speeds, demonstrating its robustness under dynamic conditions.

#### 2.2.2. Comprehensive Performance Evaluation of Specialty Grease

A systematic evaluation of the specialty grease’s properties is summarized in Table 2. The experimental results show that the grease exhibits an ultra-soft texture of NLGI Grade 0 classification (penetration: 355/0.1 mm), ensuring high fluidity and permeability for lubrication in complex mechanical systems. In terms of high-temperature stability, the dropping point of 190 °C surpasses industry benchmarks (≥180 °C), confirming colloidal stability under sustained high-temperature conditions. The four-ball friction tests measured a wear scar diameter of 0.48 mm, which not only meets the GB/T 3142 [29] (standard (≤0.60 mm) but also achieves the L-XBCHB 2 grade per ISO 12925-1 [30], validating the efficacy of its sulfur-phosphorus additive system in reducing boundary lubrication wear. The copper strip corrosion test showed a 1a level evaluation result, which is in line with the SH/T 0331 specification, indicating that the product has good compatibility with copper alloy components. The evaporation loss (1.05%) is 47.5% lower than the DIN 51817 [31] standard (≤2.0%), indicating that the base oil molecular weight distribution is reasonable and has both low volatility and high temperature durability. Through the metal plate fluidity test, 50 °C was determined as the optimal heating temperature for this specialty grease, as shown in Table 3. Collectively, these properties highlight the grease’s exceptional engineering applicability in high-temperature, heavy-load, and multi-metal coupling environments, fulfilling stringent requirements for long-term, maintenance-free lubrication in applications such as wire rope systems.

Through the above analysis, the specialty grease developed in this study exhibits fundamental differences from conventional lubricants in composition, physical state, and performance. In terms of composition, it utilizes a synthetic hydrocarbon base oil (60–65%) combined with a calcium-based metallic soap thickener (18–22%) and sulfur-phosphorus extreme-pressure additives, whereas conventional grease primarily relies on mineral oil and simpler lithium-based thickeners. Physically, the specialty grease maintains a solid-state structure at ambient temperature (Figure 1), effectively minimizing dripping and environmental contamination, in contrast to the semi-liquid state of conventional grease that often leads to uneven coating and lubricant loss. Performance-wise, the specialty grease demonstrates superior thermal stability (dropping point: 190 °C vs. ~180 °C), a 46.7% lower friction coefficient (0.08 vs. 0.15), and a 33.3% reduction in wear scar diameter under 392 N loads. Furthermore, its controlled viscosity-temperature dependence ensures optimal fluidity during application while retaining robust adhesion under operational stresses. These distinctions are systematically summarized in Table 2. Collectively, these properties highlight the grease’s exceptional engineering applicability in high-temperature, heavy-load, and multi-metal coupling environments, fulfilling stringent requirements for long-term, maintenance-free lubrication in applications such as wire rope systems.

## 3. Design of Wire Rope Intelligent Lubrication System

**Figure 5:**
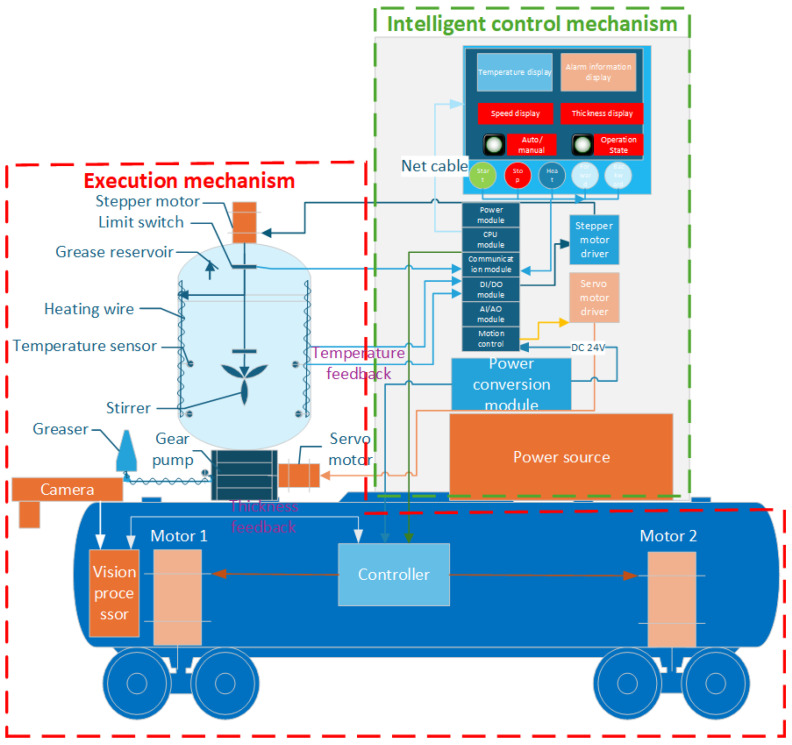
Intelligent lubrication system for wire rope.

The intelligent lubrication system developed in this study adopts a modular mobile platform design, utilizing an automatic control vehicle as the carrier chassis. It integrates an electromechanical-hydraulic integrated control system to achieve automated greasing operations. As shown in Figure 5, the system architecture primarily consists of two major components: the execution mechanism and the intelligent control system. The execution mechanism includes process control unit (heating wire, temperature sensor, limit switch), hydraulic drive unit (gear pump, grease reservoir), motion execution unit (stepper motor, servo motor), and greasing/scraping unit (grease applicator). The intelligent control mechanism is centered on a CPU module, enabling multi-device coordinated control through I/O modules and communication modules. It is equipped with dedicated driver modules (stepper/servo drivers), a human–machine interface (HMI touchscreen), and a vision processor system (industrial camera). Combined with power conversion modules, these components form a comprehensive intelligent control network, as depicted in Figure 5.

The system operates under a closed-loop control strategy, employing a Proportional-Integral-Derivative (PID) algorithm to achieve precise temperature regulation of the grease reservoir. A servo-driven system governs the gear pump to ensure quantitative grease delivery. The automatic control vehicle implements dynamic motion control in both direction and speed through PWM speed regulation technology. Simultaneously, an integrated machine vision system performs real-time thickness measurement of the grease coating. The feedback data is processed by the visual processor and transmitted to the central control unit, enabling dynamic adjustment of greasing parameters. The human–machine interface (HMI) not only provides real-time display of operational parameters (temperature, flow rate, velocity, etc.) but also facilitates data exchange with host computers via Modbus protocol. This configuration establishes an intelligent lubrication system incorporating status monitoring, fault diagnosis, and process parameter self-correction capabilities, thereby significantly enhancing coating precision and process controllability.

### 3.1. Process Control Unit

**Figure 6:**
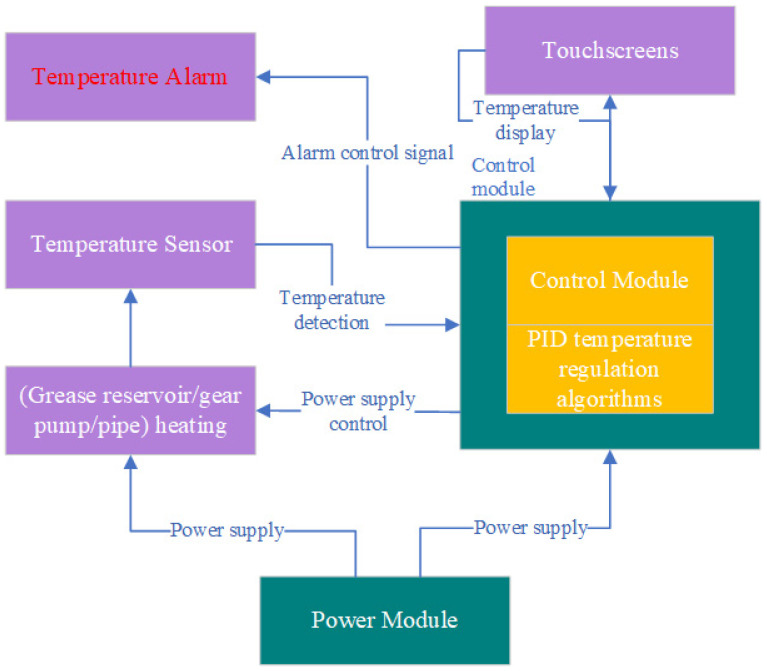
Process control unit.

The application of specialty grease requires preheating treatment to reduce viscosity and enhance fluidity. This study designed an intelligent temperature-controlled greasing system to achieve efficient and stable grease melting and delivery. The system employs a modular heating structure, where flexible heating wire networks are arranged between the inner/outer walls of the grease reservoir, within the insulation shell of the gear pump, and along the exterior of grease delivery pipes. These networks achieve three-dimensional uniform heating through enveloping thermal conduction, tailored to the geometric characteristics of each component. The core control unit integrates PID temperature regulation algorithms and establishes a closed-loop feedback system via distributed temperature sensor networks, maintaining precise grease working temperature within the set threshold range (±1.5 °C). The system automatically disconnects the heating power supply if temperatures exceed preset upper limits. Conversely, it activates compensation heating when temperatures fall below safety thresholds. It incorporates temperature alarm and safety protection functions, as illustrated in Figure 6.

### 3.2. Hydraulic Drive Unit

**Figure 7:**
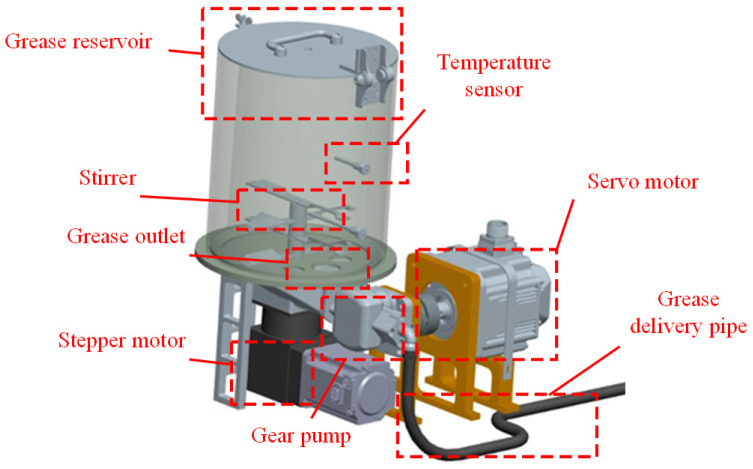
Hydraulic drive unit.

The hydraulic drive unit primarily consists of a gear pump, grease reservoir, hydraulic transmission mechanism, and motion execution unit, as shown in Figure 7. It incorporates a dual-mode operation module to enable flexible grease supply control. The system supports manual/automatic dual working modes: in automatic mode, the integrated machine vision inspection module performs real-time monitoring of grease coating quality. Based on thickness feedback signals, the control module dynamically adjusts greasing parameters to achieve closed-loop precision grease supply. In manual mode, traditional human-operated adjustment interfaces are retained to ensure operational flexibility under special working conditions. This configuration ensures adaptability to diverse application scenarios while maintaining precise control over lubrication processes.

### 3.3. Greasing and Scraping Unit

**Figure 8:**
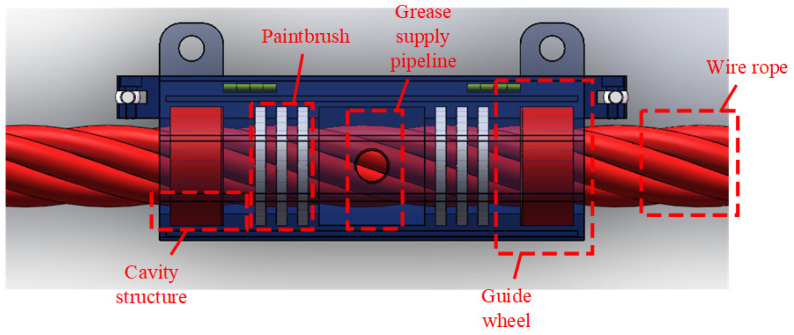
Structure of grease applicator.

The greasing and scraping unit employ a modular cavity structure with grease supply pipelines and auxiliary components. Its core component is a split-type greasing cavity featuring precision-matched upper and lower dual cavities, which integrate adjustable clamping plates and elastic seals to accommodate wire ropes with diameters ranging from 10 mm to 60 mm, ensuring stable operation across this size spectrum. During operation, the wire rope is threaded through the central through-hole of the cavity, and axial compression sealing is achieved by tightening an annular locking nut. The lubrication system, driven by a gear pump, pressurizes and delivers grease into the cavity to form a dynamic lubricating film. For non-circular wire ropes, the chain-type suspension mechanism connecting the grease applicator to the automatic control vehicle incorporates spring-loaded hinges. These hinges dynamically redistribute contact pressure along irregular surfaces, preventing grease leakage or uneven coating while maintaining uniform axial motion under constant traction. This design enables continuous and precise grease application, as illustrated in Figure 8.

### 3.4. Intelligent Control Mechanism

**Figure 9:**
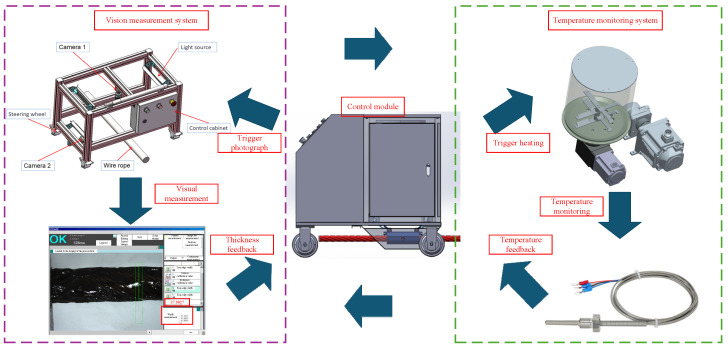
Interaction mechanism between PID and machine vision.

The intelligent control mechanism serves as the smart control core of the equipment, implementing precision regulation of the lubrication system through multimodal control strategies. The control module establishes a dual closed-loop control system based on PID algorithms. The first loop (PID) ensures the grease reservoir temperature remains within 50 ± 1.5 °C by modulating heating wire power. Simultaneously, the second loop (machine vision) captures real-time coating images, extracts thickness data via edge detection algorithms, and transmits deviation signals to the central controller. These signals trigger proportional adjustments to the greasing parameters (greasing speed, grease flow rate, and greasing time), achieving closed-loop optimization of both thermal and mechanical parameters. System status parameters are transmitted via industrial ethernet to a touchscreen display, creating a human–machine collaborative intelligent decision-making interface. This architecture ensures coordinated control and real-time process optimization, as depicted in Figure 9.

## 4. Static Analysis of Greasing Device

When the wire rope lubrication system is working normally, through kinematic analysis, the motion parameters of the lubrication device are determined, and the greasing speed, greasing thickness, and grease consumption are accurately optimized. This ensures that the greaser applies grease to the wire rope stably and evenly, achieving an ideal lubrication effect.

### 4.1. Kinematic Analysis

**Figure 10:**
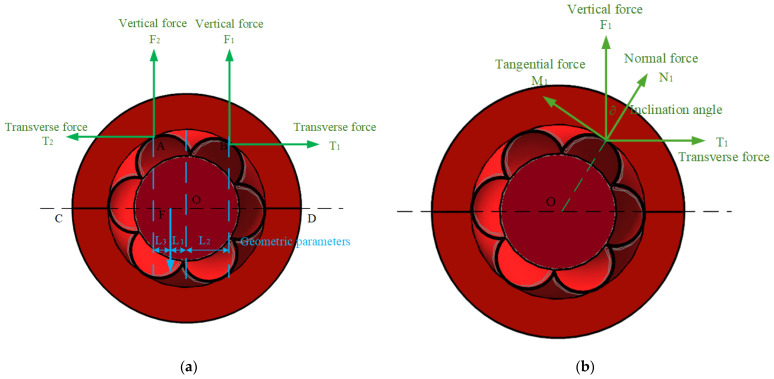
The force analysis of guide wheels: (**a**) the critical state of the wire rope lubrication device; (**b**) guide wheels contact force analysis. Green arrows indicate normal and tangential forces, while blue dashed lines represent the geometric parameters *L*_1_, *L*_2_ and *L*_3_.

Taking the operation state of the wire rope lubrication device on the horizontal wire rope as an example, it is assumed that the contact points of the lubrication device with the wire rope are symmetrically distributed on both sides of the wire rope centerline *CD*, and the guide wheels are in a critical state, that is, the edge of the wheels are separated from the surface of the wire rope. Currently, assuming that point P is the centroid of the device, the force components acting on the two guide wheels contact points are shown in Figure 10a.

Analyzing a set of guide wheels separately, the force analysis Figure 10b is obtained. Through the balance condition, the following equation can be obtained:

(2) $$N_{1}=F_{1}\cos\partial +T_{1}\sin\partial$$

(3) $$M_{1}=F_{1}\sin\partial -T_{1}\cos\partial$$

By the balance conditions, the following formula can be obtained from Figure 10b:

(4) $$F_{1}=N_{1}\cos\partial +{\mu N}_{1}\sin\partial$$

According to the moment equilibrium conditions of the lubrication device in the critical state (Figure 10a), assuming the center of mass of the device is point *F* and the guide pulleys are symmetrically distributed on both sides, the normal force *N*_1_ and tangential force *M*_1_ at the contact points between the guide pulleys and the wire rope must satisfy the following equilibrium relationships:

(5) $$\sum U_{y}=2\;(F_{1}+F_{2})=M\;(g+a)$$

(6) $$\sum V=F_{1}\left(L_{1}+L_{2}\right)=F_{2}L_{3}$$

By incorporating the friction coefficient *μ* between the guide pulleys and the wire rope, as well as the geometric parameters (*L*_1_, *L*_2_, *L*_3_), and solving the simultaneous Equations (2)–(4), the critical condition to prevent the device from slipping can be derived:

(7) $$N_{1}\geq \frac{M\;(g+a)L_{3}}{\left(L_{1}+L_{2}+L_{3}\right)\left(\mu \sin\partial +\cos\partial \right)}$$

where the denominator term μsin∂+cos∂ represents the combined influence of friction and geometric inclination angle on the normal force. Equation (7) indicates that the normal force *N*_1_ must meet a minimum value to ensure the stability of the device on the wire rope. If the friction coefficient *μ* or the guide pulley inclination angle *∂* increases, the critical normal force requirement decreases, and vice versa. The critical condition for the device not to rotate with the wire rope while in operation is as follows:

(8) $$\;(g+a)\leq \frac{r}{L_{1}}\mu \sum_{i=1}^{2}N_{i}$$

**Figure 11:**
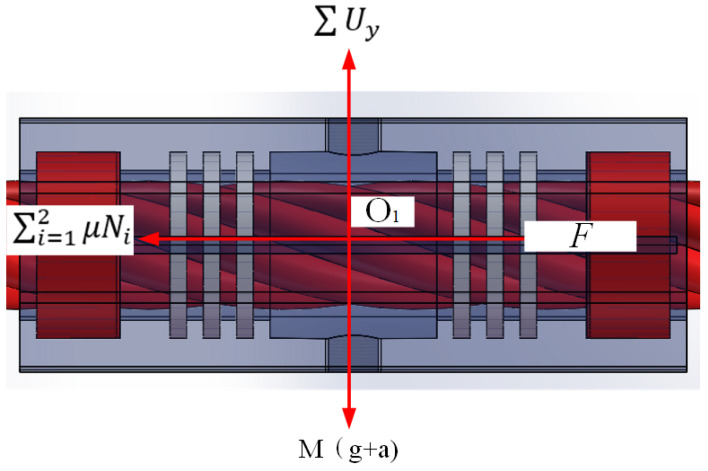
The kinematic analysis of lubrication device.

By conducting force analysis on the overall lubrication device, which is shown in Figure 11, the following formula can be obtained:

(9) $$\left\{\begin{array}{c} N_{1}\geq \frac{M\;(g+a)L_{3}}{\left(L_{1}+L_{2}+L_{3}\right)\left(\mu \sin\partial +\cos\partial \right)}\\ M\;(g+a)\leq \frac{r}{L_{1}}\mu \sum_{i=1}^{2}N_{i}\\ F\geq \mu \sum_{i=1}^{2}N_{i} \end{array}\right.$$

where *N*_1_ is normal force on the upper side of the right guide wheel’s surface, *M*_1_ is tangential force of the wire rope to the guide wheel, *F*_1_*/F*_2_ is vertical force at the point of contact between the guide wheel and the wire rope, *T*_1_*/T*_2_ is transverse force at the point of contact between the guide wheel and the wire rope, ∂ is inclination angle between the normal force and the vertical direction, *M* is total mass of the device, *g* is acceleration of gravity, *a* is acceleration of transverse vibration of the wire rope, *μ* is friction coefficient between the guide wheel and wire rope’s surface, *L*_1_*/ L*_2_*/ L*_3_ are the geometric parameters of distance from the center of mass to the vertical line of the wire rope, *r* is guide wheel radius, and *F* is the traction force of the lubrication device.

### 4.2. Grease Consumption

**Figure 12:**
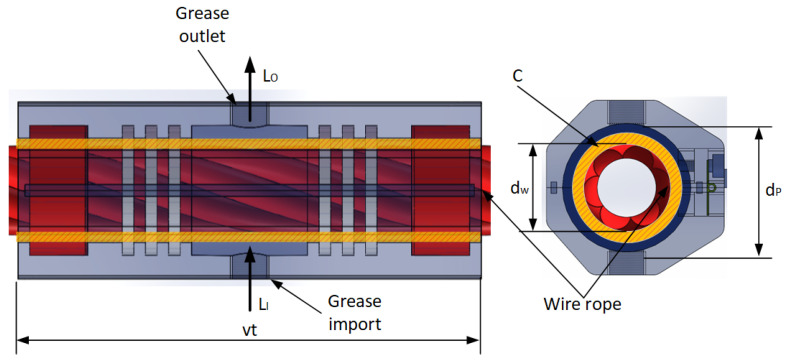
Volume consumption in straight line operation.

The calculation of grease aims to achieve adequate design and efficiency in wire rope applications. The efficiency of the lubrication process has been calculated from the second innovation of our greaser, the comparison of the magnitude of the volume of grease consumption of our design, which is able to reach optimal lengths of wire rope. The volume of grease consumption is denoted as *C*, as shown in Figure 12.

(10) $$C=\frac{\pi }{4}\frac{\left(d_{p}^{2}-d_{w}^{2}\right)}{1000}vt_{1}+L_{O}\frac{t_{1}}{60}$$

(11) $$d=\frac{d_{p}-d_{w}}{2}$$

where *t*_1_ is the greasing in time, d is the greasing thickness, *d_p_* is the diameter after greasing, *d_w_* is the diameter of wire rope, *v* is the greasing speed, *L_O_* is the grease outlet flow, and the formula for calculating the consumption of lubricating grease in straight line process is as follows:

(12) $$C=[\frac{60\pi d\left(d_{w}+d\right)v}{1000}+L_{O}]\frac{t1}{60}$$

## 5. Experimental Verification

### 5.1. Experimental Content

**Figure 13:**
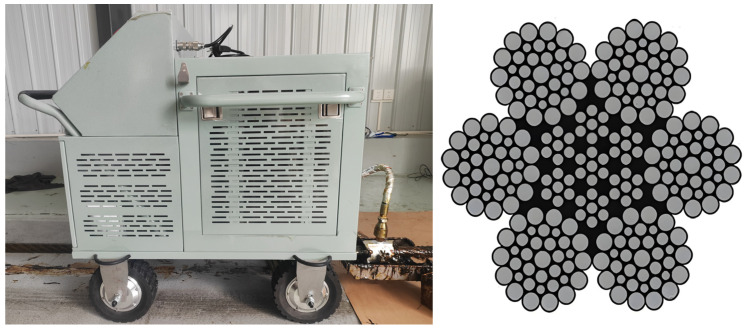
Experimental device for greasing of wire rope.

This experiment aims to establish a standardized parameter system for wire rope lubrication processes, achieving high-quality grease layer coverage through quantitative control. A 6 × 36 WS structural wire rope with a length of approximately 20 m and nominal diameter of 36.5 mm was selected as the experimental subject, with its cross-sectional morphology and experimental setup configuration shown in Figure 13. The experimental objectives include three core indicators: (1) the greasing process time should be controlled within 60 s; (2) forming a uniform thickness of the grease, where its axial thickness distribution should be stable in the 0.3–1.0 mm range; (3) achieving full coverage lubrication of the wire rope surface and rope strand gaps, with a coverage rate of 95%. The experimental process specifically includes firstly determining the greasing process parameters (greasing speed, greasing thickness, greasing flow rate) through static analysis, then inputting the determined greasing process parameters into the greasing device to perform the greasing operation, and finally using the visual measurement system to monitor the greasing thickness distribution in real-time and record the greasing parameters and effects.

### 5.2. Determination of Greasing Process Parameters

(1) Motion parameter determination

A dynamic analysis was conducted to investigate the frictional characteristics between the guide pulley and wire rope during the operation of the greasing device. Based on Equation (7), the actual traction force *F* can be calculated using the parameters provided in Table 4: mass *M*, acceleration *a*, friction coefficient *μ*, and gravitational acceleration *g*; the traction force is calculated as follows:

$$N_{1}\geq \frac{M\left(g+a\right)L_{3}}{\left(L_{1}+L_{2}+L_{3}\right)\left(\mu \sin\partial +\cos\partial \right)}\approx 119.4N$$

$$F\geq \mu \sum_{i=1}^{2}N_{i}\geq 0.6\times \left(N_{1}+N_{2}\right)=143.5N$$

Similarly, substituting other parameter combinations from Table 4 into Equation (7) yields corresponding traction forces. All calculated results are summarized in Table 4, clearly illustrating the traction force requirements under different operating conditions. This method integrates both motion acceleration and frictional resistance, providing a theoretical foundation for system design.

**Figure 14:**
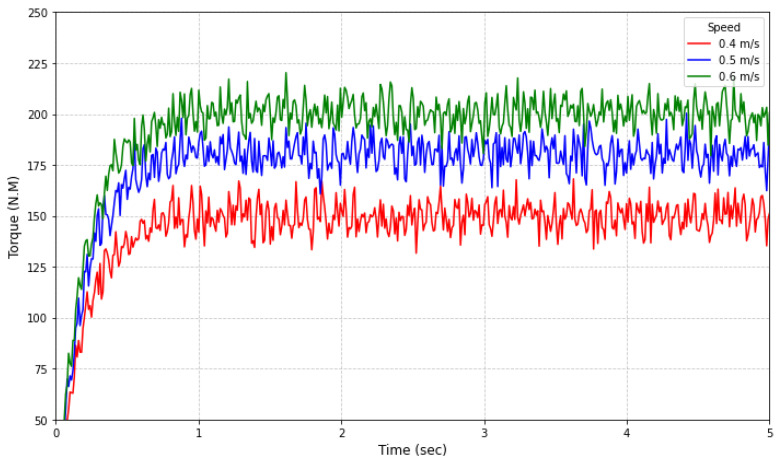
Simulation of overall motion speed of wire rope greasing device.

Using Adams 2023, a theoretical simulation model of the greasing device was established. Based on static analysis and kinematic modeling, wheel-rail constraints and driving parameters were configured to execute a 9 s/500-step forward kinematic simulation. The simulation defined static and kinetic friction coefficients as 0.3 and 0.1, respectively, with focused monitoring of torque balance characteristics under axial motion. Results demonstrate that under rolling friction-dominated conditions, the system achieves kinematic equilibrium through dynamic adjustments of traction force and torque. This process yielded critical parameters characterizing the device’s motion performance, as illustrated in Figure 14 and Table 5.

(2) Greasing parameters determination

Based on experimental content, greasing device characteristics, and simulation modeling, key parameters were determined as follows: the greasing time was $t_{1}\leq 60\;s$; the greasing thickness should be stable in the 0.3–1.0 mm range; and the grease layer needed to uniformly cover the wire rope. The specific greasing device parameters are shown in Table 6, and gear pump operational parameters are shown in Table 7.

Based on the analysis of grease performance, high-viscosity and high-consistency grease was selected for closely braided wire rope to enhance its adhesive properties and anti-extrusion performance. The grease employed in this study maintains a solid state at room temperature and demonstrates robust adhesion characteristics under normal operating conditions. Due to the poor flowability inherent to high-viscosity grease, a preheating process using the heating mechanism integrated within the grease application device was implemented prior to coating operations. Detailed technical parameters of the grease are systematically presented in Table 8.

**Figure 15:**
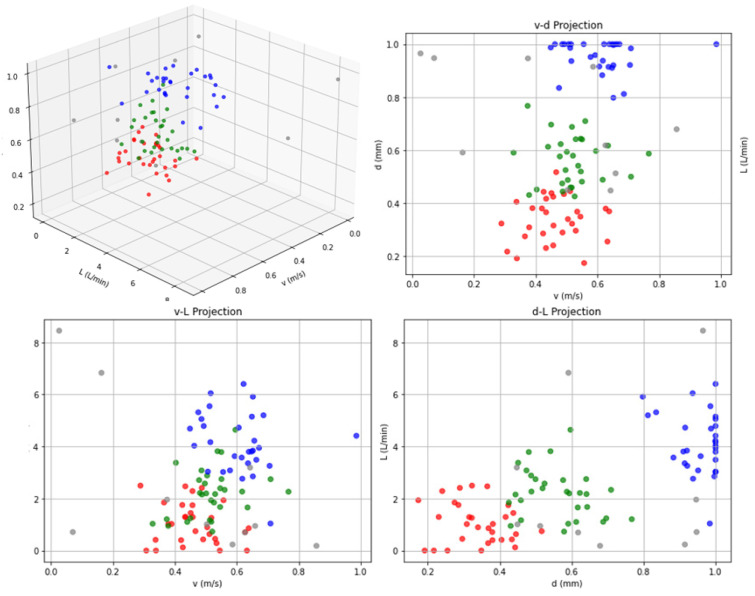
Greasing process parameters simulation calculation.

Simulation calculations were conducted using MATLAB R2023a on the grease consumption model, revealing the correlations between wire rope greasing process parameters (greasing speed, greasing thickness, and grease flow rate) and grease consumption. These quantitative relationships are graphically illustrated in Figure 15.

As evidenced by the simulation results in Figure 15 and detailed in Table 9 regarding grease application parameters; the red curve (Scenario 1) demonstrates a coating speed of 0.48 m/s, greasing thickness of 0.35 mm, grease flow rate of 0.93 L/min, application duration of 44.4 s, and grease consumption of 0.69 L. Based on the gear pump’s rated displacement of 4 mL/r specified in Table 5, the pump speed was calculated as 233 r/min. The green curve (Scenario 2) presents enhanced parameters with a coating speed of 0.52 m/s, greasing thickness of 0.57 mm, and grease flow rate of 1.92 L/min, corresponding to a pump speed of 480 r/min. The blue curve (Scenario 3) exhibits optimized performance metrics including a coating speed of 0.60 m/s, greasing thickness of 0.98 mm, and grease flow rate of 4.32 L/min, achieving a pump speed of 1080 r/min through proportional adjustment.

The simulation results derived from the theoretical framework provide critical insights into the greasing process parameters of the lubrication system. By optimizing greasing speed, greasing thickness, grease flow rate, and grease consumption, the model predicts a stable and uniform grease distribution. These findings establish a theoretical foundation for subsequent experimental validation, where the simulated parameters will be rigorously tested to evaluate their practical effectiveness in achieving precision-controlled lubrication performance.

### 5.3. Experimental Process

**Figure 16:**
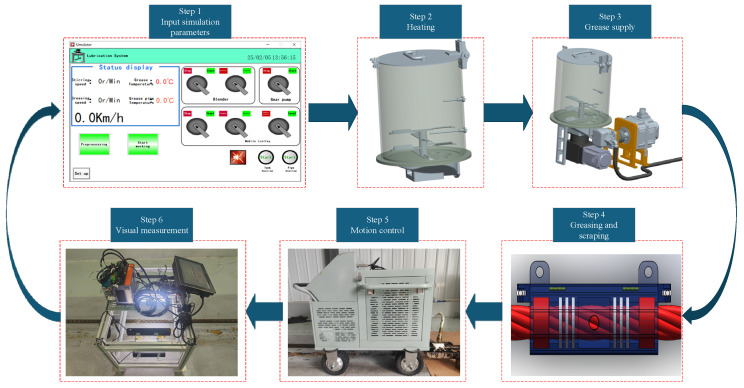
Experimental process of wire rope lubrication.

The wire rope greasing process is implemented as follows: First, the greasing process parameters obtained from the simulations (including greasing speed, greasing thickness, grease flow rate) are input into the greasing control system. Upon activation of the lubrication device, the system elevates the grease temperature to the preset level via the process control unit while the execution mechanism simultaneously activates the metering pump to initiate constant-speed coating application. During operation, the vision measurement system performs real-time scanning of the rope surface to measure greasing thickness. If deviations from the simulated greasing thickness are detected, the system automatically feeds the thickness error values back to the control unit, triggering dynamic adjustments to the greasing parameters. Through multiple iterations of the “greasing– measurement–parameter iteration” closed-loop control mechanism, adaptive lubrication control with a precision of ±0.15 mm is ultimately achieved, as illustrated in Figure 16.

### 5.4. Experimental Results

**Figure 17:**
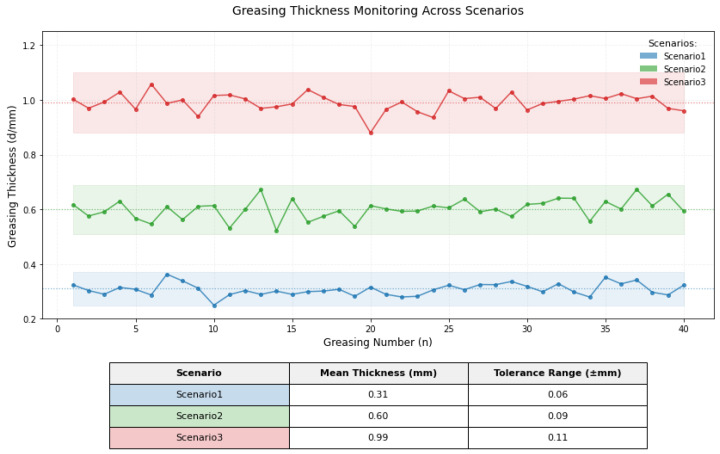
Thickness distribution of grease in three experimental scenarios (representative dataset from 40 repeated trials).

Each experimental scenario (Scenarios 1–3) was repeated 40 times under controlled conditions to ensure statistical robustness. The data presented in Table 10 represents one representative dataset from these trials, while Figure 17 aggregates all trials to illustrate thickness distribution trends. Data from repeated trials were analyzed to compute mean values, standard deviations, and 95% confidence intervals. A one-way ANOVA confirmed no significant inter-scenario variability (*p* > 0.05). All scenarios exhibit thickness distributions within their preset tolerance bands, validating the stability and repeatability of the greasing process parameters.

**Figure 18:**
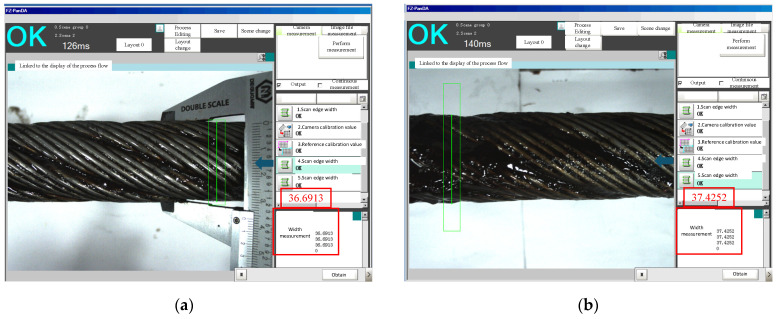

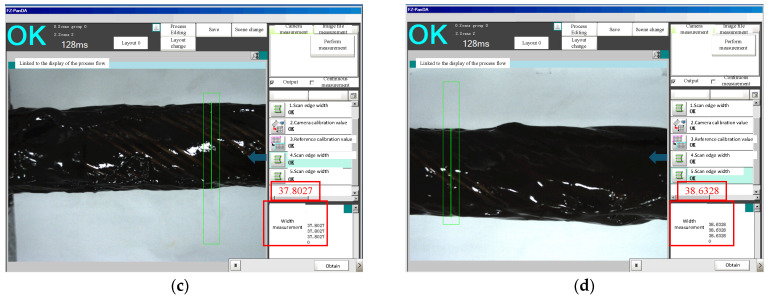
Experimental greasing effect: (**a**) original state of wire rope; (**b**) Scenario 1 0.3 mm greasing effect; (**c**) Scenario 2 0.5 mm greasing effect; (**d**) Scenario 3 1.0 mm greasing effect.

Figure 18 presents a comparative analysis of the actual greasing effectiveness under different greasing process parameters, with subfigures (b)–(d) illustrating representative outcomes from one trial selected from 40 repeated experiments. Subfigure (a) displays the baseline diameter measurement results of the wire rope without greasing process implementation, while subfigures (b)–(d) correspond to Scenario 1–3 greasing parameters, respectively. Full statistical distributions of all trials are provided in Figure 17. At the maximum tested speed of 0.6 m/s, the grease layer thickness remained within 0.3–1.0 mm (±0.15 mm), with no observable film rupture or uneven distribution, validating the system’s adaptability to high-speed operations.

Comparative analysis between experimental results and theoretical simulations demonstrates that all three greasing scenarios achieved the targeted greasing performance. The measured mean thickness values are 0.31 ± 0.05 mm (Scenario 1), 0.60 ± 0.08 mm (Scenario 2), and 0.99 ± 0.10 mm (Scenario 3), aligning closely with theoretical predictions. Grease thickness deviations are controlled within ±0.15 mm, validating the capability of the developed intelligent lubrication system to meet wire rope lubrication requirements, thereby confirming its design objectives.

The experimental results and comparative analysis demonstrate that the designed wire rope maintenance device achieves significant improvements over existing systems in Table 11. Compared to conventional hoist-based methods [22,23,24,25,26], our design reduces traction force by approximately 30%, while directional scraping and sealed lubrication mechanisms cut grease waste by 15–20% relative to spray lubrication techniques [23]. The device adapts to varying rope diameters, offering a practical, efficient, and environmentally friendly solution for industrial wire rope maintenance.

## 6. Conclusions

This study developed an innovative on-line intelligent lubrication system for wire ropes using a specialty grease formulation to address the limitations of conventional lubrication methods. Comprehensive analysis of the specialty grease revealed superior tribological properties through SEM, FT-IR, and four-ball friction tests, demonstrating a 46.7% reduction in average friction coefficient and 33.3% smaller wear scar diameter compared to conventional grease under 392 N loading. The modular lubrication system integrated a temperature-regulated process control unit, dual-mode hydraulic drive, greasing-scraping mechanism, and an intelligent control framework employing dual closed-loop PID control with machine vision monitoring. Experimental validation on a 20 m long, 36.5 mm diameter wire rope confirmed system efficacy, achieving full lubrication coverage within 60 s with controlled grease thickness (0.3–1.0 mm). Key innovations include the novel application of high-performance grease with enhanced thermal stability/adhesion and the system’s adaptive architecture enabling real-time process optimization.

Future research will focus on advancing both the grease formulation and system capabilities. To further improve the temperature stability of the specialty grease, efforts could target the optimization of thickener and additive systems. For instance, incorporating hybrid thickeners such as lithium-calcium composite soaps with higher crystallinity may strengthen the three-dimensional network structure, thereby improving resistance to thermal degradation. Additionally, employing higher-molecular-weight synthetic base oils could reduce volatility and enhance oxidative stability at elevated temperatures. Advanced additives, such as nano-ceramic particles, may further mitigate thermal thinning by reinforcing the grease matrix. These strategies aim to extend the operational temperature range beyond 200 °C while maintaining adhesion and anti-wear performance. Concurrently, future work will expand the system’s adaptability to diverse rope geometries and operational speeds, alongside integrating advanced sensing technologies for real-time wear and degradation monitoring. This research establishes a foundational framework for intelligent lubrication technologies, offering significant potential to enhance safety and reliability in wire rope-dependent industrial applications.

## Figures and Tables

**Table 2:** Performance comparison between specialty grease and conventional grease.

Test Item	Specialty Grease	Conventional Grease	Standard Requirement	Conclusion
Worked penetration (0.1 mm)	355	310–330 (NLGI Grade 1)	NLGI Grade 0 (335–385)	↑ Ultra-soft texture, enhances penetration into wire strands.
Dropping point (°C)	190	160–180	≥180	↑ Thermal stability (exceeds conventional by 10–30 °C).
Four-ball WSD (mm)	0.48	0.65–0.80	≤0.60	↓ 33% smaller wear scar, superior EP/anti-wear performance.
Copper strip corrosion	Class 1a	Class 2–3	No green discoloration	↑ Metal compatibility, minimal corrosive effects.
Evaporation loss (%) (180 °C)	1.05	2.0–3.5	≤2.0 (typical)	↓ 47% lower loss, reduced environmental contamination.

**Table 3:** Specialty grease metal plate fluidity test.

Serial Number	Temp	Thickness of Grease Application	Travel Distance (Maximum 60 mm)
40 °C-1	40 °C	1 mm	0 mm
40 °C-2	40 °C	1 mm	0 mm
40 °C-3	40 °C	3 mm	0 mm
40 °C-4	40 °C	3 mm	0 mm
50 °C-1	50 °C	1 mm	0 mm
50 °C-2	50 °C	1 mm	0 mm
50 °C-3	50 °C	3 mm	6 mm
50 °C-4	50 °C	3 mm	4 mm
60 °C-1	60 °C	1 mm	3 mm
60 °C-2	60 °C	1 mm	5.5 mm
60 °C-3	60 °C	3 mm	60 mm
60 °C-4	60 °C	3 mm	60 mm
70 °C-1	70 °C	1 mm	60 mm
70 °C-2	70 °C	1 mm	60 mm
70 °C-3	70 °C	3 mm	60 mm
70 °C-4	70 °C	3 mm	60 mm
**Test** **P** **rotocol**	1. Apply the sample grease to the designated area of the metal plate with specified thicknesses (1 mm and 3 mm). 2. Place the grease-coated metal plate into an oven and maintain constant temperatures at 40 °C, 50 °C, 60 °C, and 70 °C for 15 min. 3. Immediately remove the metal plate from the oven, position it vertically, and measure the flow distance of the grease within 1 min.		

**Table 4:** Actual traction force calculation results.

*a* (m/s^2^)	*M* (Kg)	*μ*	*g* (m/s^2^)	*L*_1_ + *L*_2_ + *L*_3_ (mm)	*L*_3_ (mm)	∂ (°)	*N*_1_ (N)	*F * (N)
0.64	20	0.6	9.8	30	20	30	119.4	143.5
2.05	20	0.6	9.8	30	20	30	135.5	162.6
3.72	20	0.6	9.8	30	20	30	154.6	185.5

**Table 5:** Experimental parameters of traction of wire rope greasing device.

Startup Speed	Traction Force
0.4 m/s	148.7 N
0.5 m/s	177.6 N
0.6 m/s	201.3 N

**Table 6:** Specific greasing device parameters.

*v_min_* (m/s)	*v_max_* (m/s)	*L_min_* (L/min)	*L_max_* (L/min)	*t*_1*max*_ (s)	*vt*_1_ (m)	*d_w_* (mm)
0	1	0	10	60	20	36.5

**Table 7:** Gear pump operational parameters.

Discharge Volume (cc/rev)	Pressure (kgf/cm^2^)		Speed (rpm)	Weight (kg)	Remarks
4	Work	Tallest	800–4500	1.67	/
	210	250			

**Table 8:** The technical parameters of the grease.

Base Oil	Thickener	Dropping Point (°C)	Worked Penetration (0.1 mm)	Adhesion (L/min)	Preheating Temperature (°C)
Synthetic hydrocarbon	Metallic soap	190	355	High	50

**Table 9:** Operational parameters of greasing device in simulated scenarios.

Scenario	Greasing Time (s)	Greasing Speed (m/s)	Greasing Thickness (mm)	Grease FLOW Rate(L/min)	Greased Diameter (mm)	Grease Consumption (L)	Grease Supply Speed (r/min*)*
1	44.4	0.48	0.35	0.93	37.2	0.69	233
2	39	0.52	0.57	1.92	37.54	1.25	480
3	33.6	0.60	0.98	4.32	38.46	2.42	1080

**Table 10:** Experimental results.

Scenario Number	Greasing Time (s)	Greasing Speed (m/s)	Greased Diameter (mm)	Thickness of Greasing (mm)	Grease Supply Speed (r/min*)*
1	35	0.57	37.4252	0.37	300
2	38	0.52	37.8027	0.56	500
3	36	0.55	38.6328	0.97	1200

**Table 11:** Performance comparison of the proposed device with the existing systems.

Metric	Proposed Device	Hoist-Based Systems [22]	Spray Lubrication [23]	Robotic Platforms [24,25,26]
Traction force	<200 N	>300 N (inferred from [22])	N/A	200–300 N (motor-driven)
Operational speed	0.6 m/s	0.3–0.5 m/s (manual)	N/A	0.4–0.8 m/s
Lubricant consumption	Low (directional scraping + sealed chamber)	High (inefficient scraping)	High (15–20% waste)	Moderate (module-dependent)

## Data Availability

Data are contained within the article.
